# Randomized controlled trial on feedback-informed internet-delivered psychodynamic therapy for adolescents with depression: A trial that failed to recruit enough participants

**DOI:** 10.1016/j.invent.2026.100923

**Published:** 2026-02-22

**Authors:** Björn Philips, Jakob Mechler, Carl-Johan Uckelstam, Gerhard Andersson, Per Carlbring, Julian Edbrook-Childs, Fredrik Falkenström, Robert Johansson, Peter Lilliengren, Katja Lindert Bergsten, Nick Midgley, Rolf Sandell, Agneta Thorén, Naira Topooco, Randi Ulberg, Karin Lindqvist

**Affiliations:** a Department of Psychology, Stockholm University, Stockholm, Sweden; b Department of Psychology, Uppsala University, Uppsala, Sweden; c Department of Behavioural Sciences and Learning, Department of Biomedical and Clinical Sciences, Linköping University, Linköping, Sweden; d Department of Clinical Neuroscience, Karolinska Institutet, Stockholm, Sweden; e Department of Health, Education and Technology, Luleå University of Technology, Luleå, Sweden; f School of Psychology, Korea University, Seoul, South Korea; g Department of Clinical, Educational and Health Psychology, University College London, UK; h Evidence Based Practice Unit, Anna Freud National Centre for Children and Families, London, UK; i Department of Psychology, Linnaeus University, Växjö, Sweden; j Child Attachment and Psychological Therapies Research Unit (ChAPTRe), UK; k Department of Psychology, Lund University, Lund, Sweden; l The Erica Foundation, Stockholm, Sweden; m University of Oslo, Oslo. Norway

**Keywords:** Adolescent depression, Internet-delivered psychotherapy, Psychodynamic therapy, Feedback-informed treatment, Adaptive intervention, Recruitment challenges, Digital mental health

## Abstract

**Background:**

Internet-delivered psychodynamic therapy (IPDT) has been found to be effective for adolescents with depression in previous randomized controlled trials. The present study aimed to evaluate an adaptive, feedback-informed version of IPDT, designed to improve outcomes for participants identified early as at risk of non-response.

**Methods:**

A randomized controlled trial targeting adolescents aged 15–19 years with mild to moderate major depressive disorder. Participants were recruited through social media, national and local advertising, schools, and user organizations. After three weeks of standard IPDT, participants classified as at risk by a prediction algorithm were randomized to either adapted or standard treatment. The planned sample size was 240 participants. Despite extensive nationwide recruitment efforts during 2024, only 35 participants were enrolled before the study was discontinued.

**Results:**

Recruitment difficulties were primarily due to recent European Union regulations prohibiting profiling-based online advertising for minors, which eliminated access to previously effective social media recruitment channels. Participants who completed treatment showed significant pre- to post-treatment improvements in depressive symptoms (*d* = 1.08), anxiety (*d* = 0.74), and emotion regulation (*d* = 0.79). The predictive algorithm showed promising results in classifying patients as responders or non-responders.

**Conclusions:**

Although the trial was underpowered, the findings provide promising within-group effects and valuable lessons for future digital mental health research involving minors. New recruitment infrastructures that comply with data protection laws are needed to ensure feasibility of online psychotherapy trials. Continued development of adaptive, feedback-informed IPDT for adolescents with depression is needed.

**Trial registration:**

ClinicalTrials.gov Identifier: NCT06193772

## Highlights

• This randomized controlled trial was designed to evaluate a feedback-informed version of internet-delivered therapy.
• A predictive algorithm had been developed to identify participants at risk of non-response after three weeks of treatment.
• The trial met challenges in recruiting participants, due to recent EU regulations against social media ads for minors.
• Hence, only 35 participants were included, instead of the planned sample size of 240 participants.

## 1 Introduction

Depression during adolescence is common and associated with a range of long-term difficulties, including recurrent depression, impaired functioning, and elevated suicide risk (Alaie et al., 2019; Mörk et al., 2014). Despite these serious consequences, only a minority of affected adolescents receive adequate treatment (Rocha et al., 2015). Improving early access to psychological interventions is therefore a pressing public health concern.

Internet-delivered psychological treatments have shown efficacy comparable to traditional face-to-face therapies for adults (Hedman-Lagerlöf et al., 2023) and promising effects in young populations (López-Soler et al., 2024). Internet delivery can reduce barriers to care, such as stigma, geographical limitations, and waiting times (Andersson et al., 2019) and thus has the potential to reach adolescents who might otherwise avoid treatment (Foroushani et al., 2011; Salzer et al., 2018).

Although evidence-based therapies such as cognitive behavioural therapy (CBT) seem to be effective for children and adolescents with depression (Oud et al., 2019), a substantial proportion of patients do not respond satisfactorily. Recent meta-analyses found that response rates for children and adolescents receiving psychotherapy on average were below 40%, and differences compared to control groups often disappear at follow-up (Cuijpers et al., 2021; Weersing et al., 2025). These findings highlight the need for treatment alternatives and for improved methods to identify, and support non-responders during treatment (Rozental et al., 2017).

Psychodynamic psychotherapy (PDT) is one such alternative. Meta-analyses suggest comparable effects to other therapies for youth with mood and anxiety disorders (Abbass et al., 2013). Internet-delivered PDT (IPDT) has shown promising effects for adults (Lindegaard et al., 2020) and, in our prior trials within the ERiCA project, for adolescents with depression. A smaller RCT (*n* = 72) demonstrated the efficacy and feasibility of IPDT (Lindqvist et al., 2020), and a subsequent non-inferiority trial (*n* = 272) found IPDT to be comparable to internet-based CBT (Mechler et al., 2022).

Although effective, both IPDT and ICBT showed response rates slightly under 50% in the non-inferiority trial (Mechler et al., 2022). This pointed to the need to explore adaptive treatment strategies aimed at reducing non-response. Using data from the non-inferiority trial, we developed an algorithm to identify participants at risk of non-response early in treatment. The present randomized controlled trial was designed to evaluate whether adapting IPDT for such participants could improve outcomes. However, despite careful planning, the study encountered major recruitment difficulties, leading to insufficient sample size at termination of the trial.

### 1.1 Aims and study design

This trial was the third study in the ERiCA project, which aims to develop and evaluate cost-effective internet-delivered treatments for adolescent depression. The specific goal of the present trial was to test an adaptive treatment strategy in internet-delivered psychodynamic therapy (IPDT). Based on data from a previous large RCT (Mechler et al., 2022), an algorithm predicting non-response after three weeks of treatment was used to identify participants at risk. These participants were to be randomized either to an adapted treatment arm or to continue with standard IPDT.

The primary research questions concerned whether adapted treatment could reduce the risk of non-response among at-risk participants, and whether non-response could be accurately predicted early in treatment. Secondary aims were to explore predictors, moderators, and mediators of treatment response.

Despite a well-developed study design, recruitment proved to be a major challenge. Out of a planned sample of 240 participants, only 35 adolescents were enrolled, followed by the decision to discontinue the trial in accordance with time plan. The following report describes the recruitment process, explores potential reasons for the difficulties encountered, and lessons learned for future clinical trials targeting adolescent depression.

## 2 Methods

### 2.1 Study setting

The study was conducted at Stockholm University in collaboration with Linköping University. Treatment was delivered entirely online via a secure platform previously used in several internet-based psychotherapy trials (Vlaescu et al., 2016). The nationwide recruitment strategy was intended to enable broad participation and increase sample diversity with respect to geography and socioeconomic background. The trial was pre-registered at ClinicalTrials.gov (NCT06193772) and was approved by the Swedish Ethical Review Authority (date 2023-11-13, reg.nr. 2023-05530-01). In accordance with Swedish law, no parental consent was required for adolescents aged ≥15 years.

### 2.2 Eligibility criteria

Adolescents aged 15–19 years with mild to moderate major depressive disorder (MDD) according to DSM-5 were eligible. Diagnoses were established through a structured interview (MINI 7.0; Sheehan et al., 1998) over telephone. Exclusion criteria included elevated suicidality, concurrent psychological treatment, unstable medication, psychotic or bipolar disorder, antisocial personality disorder, autism spectrum disorder, and substance abuse. Participants needed internet access and sufficient proficiency in Swedish.

### 2.3 Intervention

All participants received internet-delivered psychodynamic therapy (IPDT), consisting of eight therapist-supported modules over ten weeks (Mechler et al., 2024). Modules contained texts and videos followed by written exercises with individualized therapist feedback. The program aimed to reduce emotional avoidance, increase affect awareness, and improve emotional regulation and relational functioning. Content and examples were adapted for adolescents, with attention to developmental aspects relevant to this age group.

Therapists were advanced clinical psychology students under weekly supervision by licensed psychologists and treatment developers. They received a one-day training in IPDT and had continuous access to participants' weekly progress data, allowing for monitoring and reminders in case of inactivity.

### 2.4 Adapted treatment

At week 3, an algorithm developed from prior ERiCA data (Mechler et al., 2022) identified participants at risk for non-response. These participants were to be randomized to either continue standard IPDT or receive an adapted treatment. In adapted treatment, therapists followed a manual, where they suggested modifications such as weekly synchronous chat sessions, weekly/biweekly phone calls, reminders to partake in treatment, or shorter modules. Adaptations were decided collaboratively between therapist and participant to improve engagement and progress, and therapist could discuss this in a chat session or a phone call.

### 2.5 Outcomes and measures

The primary outcome was treatment response, operationalized using the Proportion Improvement (PI) method (Hiller et al., 2012). The PI method quantifies the proportion of “true change” relative to the “possible change,” requiring ≥50% improvement within the clinical range and ≥25% improvement overall on the Quick Inventory of Depressive Symptomatology—Adolescent Self-Report (QIDS-A17-SR; Bernstein et al., 2010) measuring depressive symptoms. Secondary outcomes included anxiety using Generalized Anxiety Disorder 7-item scale (GAD-7; Spitzer et al., 2006) and emotion regulation using Difficulties in Emotion Regulation Scale 16-item scale (DERS-16; Bjureberg et al., 2016). Measures were administered online at baseline and post-treatment, and for QIDS-A17-SR also weekly during treatment. For an entire list of the measures used in the study, see the trial registration on Clinicaltrials.gov.

### 2.6 Procedure and randomization

**Fig. 1:**
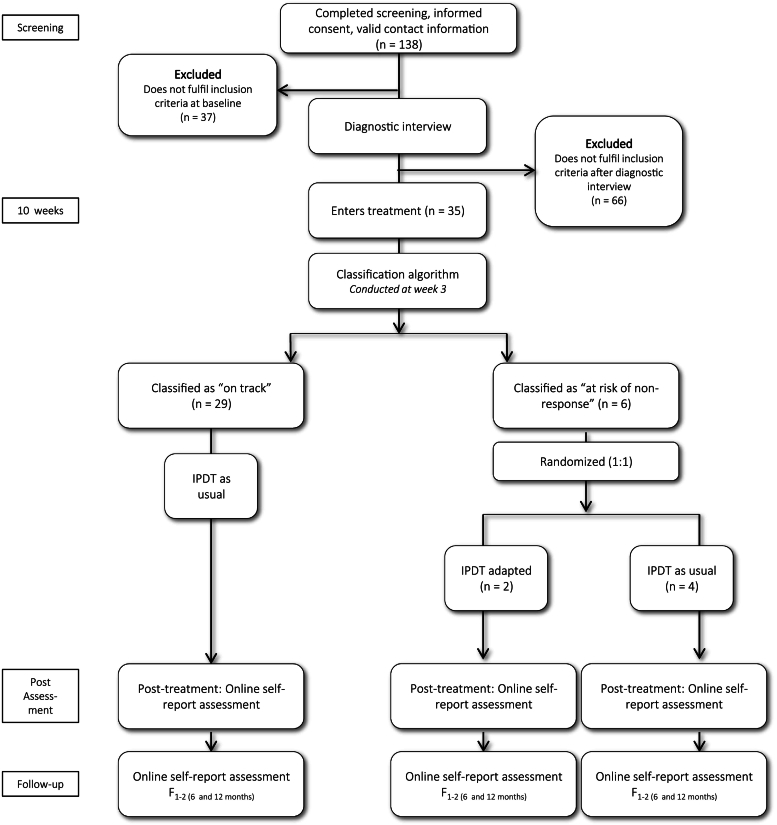
Trial profile.

As illustrated in Fig. 1, participants accessed study information and consented online before completing screening questionnaires and a diagnostic interview. Eligible adolescents were enrolled in standard IPDT. After three weeks, those classified as at risk by the prediction algorithm were to be randomized (1:1) to adapted or standard IPDT by an independent researcher using computerized randomization.

### 2.7 Recruitment strategy and sample characteristics

Power analysis using Sealed Envelope (2012) indicated a required sample of 240 participants to detect expected differences between conditions with 80% power, assuming a 20% attrition rate. Recruitment was planned via social media, national and local advertising, schools, and user organizations. We adhered to the recruitment strategy that had demonstrated high effectiveness in previous studies in the project (Lindqvist et al., 2020; Mechler et al., 2022). The project had accounts on Instagram, Facebook and Tiktok where we continuously made postings and uploaded new videos. We contacted several Swedish influencers and celebrities, and some of them agreed to spread information about the study. We used a list, developed throughout the project, of about 1.200 persons who work in Swedish youth centers, driving schools, health centers, youth clinics, etc. and emailed them information about the study. In these emails we enclosed digital materials such as videos and posters, but we also offered to send physical materials such as flyers, business cards and posters. We also had information about the study on a Swedish website designed for study information involving internet-delivered treatments (www.studie.nu). When we used this recruitment strategy in our non-inferiority RCT during three semesters 2019–2020, it resulted in 996 registered adolescents leading to 272 included participants (Mechler et al., 2022).

In the years preceding the present study, the European Union (EU) made several decisions to protect minors (under 18 years) from profiling-based advertising in social media. The General Data Protection Regulation (GDPR) from May 2018 laid the groundwork of data protection including the processing of children's data. The revised Audiovisual Media Services Directive (AVMSD) from November 2018 added explicit restrictions about that personal data from minors must not be processed for commercial processes such as direct marketing, profiling and behaviorally targeted advertising. The Digital Services Act (DSA) from October 2022 states that online platforms shall not present advertisements based on profiling when they are aware with reasonable certainty that the recipient is a minor. Under this EU regulatory pressure, Meta (owner of Instagram and Facebook) introduced some changes to limit how advertisers could target teens in January 2023. This was followed by an independent review by the European Data Protection Board (EBPB) further pressuring Meta to change advertising policy, followed by decision by the Irish Data Protection Commission in November 2023 to ban Meta's advertising for minors. After the above sequence, Meta's business/ads documentation and product behavior reflect that advertisers cannot deliver ads to audiences aged under 18 in the EU, noted as effective in November 2023.

Recruitment for the present trial was hampered by several challenges, which will be further described in the Results section. Of the 138 adolescents who completed screening, 97 were excluded due to following reasons: 20 could not be reached, 10 declined participation, 6 not depressed (QIDS-A17-SR <9), and 61 were excluded after diagnostic interview (6 not depressed, 25 were in ongoing treatment, and 35 fulfilling other exclusion criteria). Hence, 41 adolescents were offered participation, although 6 declined or did not respond. Ultimately, only 35 adolescents were enrolled in the study after one year (2024) of intensive recruitment efforts. Participants were predominantly of female gender (74%) with average age *M* = 17.7. Most frequent co-morbid disorders were anxiety disorders. Further demographic and diagnostic data on participants are presented in Table 1.

**Table 1:** Demographic data at baseline.

Characteristic	Total sample (*n* = 35)
**Gender, n (%)**	
Female	26 (74.3)
Male	8 (11.4)
Uncertain or other	1 (5.7)
**Age (years), M (SD)**	17.66 (1.47)
**Years from first depressive episode, M (SD)**	4.31 (3.01)
**Diagnosis, n (%)**	
Major depressive disorder	35 (100)
Panic disorder	5 (14.3)
Agoraphobia	2 (5.7)
Social anxiety disorder	10 (28.6)
Generalized anxiety disorder	7 (20.0)
Post-traumatic stress disorder	3 (8.6)
Obsessive-compulsive disorder	4 (11.4)
Binge eating disorder	3 (8.6)

### 2.8 Algorithm development

Data from Mechler et al. (2022) were used to develop the algorithm for identifying participants at risk of non-response or clinical deterioration. All available data collected from pre-treatment through treatment week 3 (see Mechler et al., 2020, for a complete list of variables) were considered in developing the algorithm. Several different statistical models were trained using standard supervised learning procedures including stratified 5-fold cross-validation. The training dataset included 207 patients (80%) of the sample in Mechler et al., 2022), and the test dataset included 54 patients (20%). The overall non-response rate in the sample was 37%.

The evaluated classification algorithms included *Random Forest* (RF), *Naïve Bayes* (NB), and a *Generalized Linear Model with Elastic Net Regularization* (GLM-ENR). In addition, a novel approach based on a mixed-effects regression model implemented within a supervised learning framework was explored. Classification algorithms were optimized using model-specific hyperparameters (e.g., *mtry*, *lambda*, *laplace*). The mixed models were estimated using a Bayesian approach with Markov Chain Monte Carlo (MCMC) sampling, following the procedure described by Rizopoulos (2016). Specifically, several model specifications were tested on the training data, each including weekly QIDS-A17-SR scores from pre-treatment through week 3, along with different baseline covariates and various change terms (e.g., linear, quadratic, logarithmic). MCMC sampling made it possible to collect each models parameters and predict each participant's post-treatment score, which in turn was compared against the IP criterion to classify individuals as at risk of non-response or not.

Model performance for all models were assessed using the F1 measure, which balances precision (*the proportion of correctly predicted non-responders among all predicted non-responders*) and recall (*the proportion of correctly predicted non-responders among all actual non-responders*) into a single metric (Bishop, 2006). The model with the highest F1 score was selected as the optimal model. To evaluate robustness, the final model derived from training was tested on unseen data from the test set.

The best-performing and most stable model for identifying at-risk participants was a mixed-effects model with a logarithmic change term for weekly QIDS-A17-SR scores, and baseline PID-5-BF as covariate. This model achieved an F1 score of 0.65 during training and 0.68 when applied to the test set. It correctly identified 80% of non-responders (sensitivity) and 68% of responders (specificity), resulting in a balanced accuracy of 74%.

In contrast, therapists participating in the study were asked to identify their own participants at risk of non-response at the same treatment week. Their predictions yielded an F1 score of 0.52, with a sensitivity of 50%, specificity of 65%, and a balanced accuracy of 58%.

### 2.9 Analyses

Our original plan for outcome analysis was that primary outcomes would be assessed using odds ratios. Secondary outcomes exploring trajectories of change was planned as using rates-of-change estimates based on linear mixed models. Differences in efficacy between conditions was planned to be investigated by modeling interaction effects of group and time. These methods have been recommended for RCTs investigating internet interventions (Hesser, 2015). Given the limited sample size, these analyses could not be conducted as originally intended. Thus, we only analyzed outcome in terms of *t*-tests for dependent samples and within-group effect sizes for the total sample using SPSS version 29.

## 3 Results

### 3.1 Recruitment process

The first period for recruitment and enrollment lasted from January to March 2024, to ensure that all 10-week treatments would be terminated during the semester. When we started recruitment, the new rule from Meta was recently enforced (November 2023), prohibiting us from directing advertisements on Instagram and Facebook to adolescents aged 15–17. We received legal advice that confirmed our view that information about the study should be regarded as research information and not advertising. A long series of discussions with Meta representatives followed, on telephone and text chat, to try to persuade them to recognize our interpretation and permit the dissemination of our information campaign accordingly. After a long stretch of contradictory responses from Meta, no information to minors was allowed to run. Instead, we posted videos/pictures on Instagram for adolescents aged 18–19, as well as on Facebook for adults who might know adolescents who struggled with depression. In the present study, we have made several postings each week on social media. In addition, we used other recruitment methods, such as spreading project information through influencers and celebrities, sending emails to about 1.200 persons who are working with adolescents, appearing ourselves in podcasts talking about the project, and spreading project information through the communicators at Stockholm University.

During the first period of recruitment and enrollment January–March, we received 106 registrations from adolescents, which ended in 22 included participants and 40 exclusions.

The second period of recruitment and enrollment lasted from August to October 2024, again to ensure that all 10-week treatments would be finished during the semester. To improve enrollment, we employed a research assistant who spread project information through telephone calls and emails. In addition, project information was shared at several events focusing on mental ill-health among young people. We continued with several further discussions with Meta representatives, without result. Despite all our efforts, enrollment was even slower during the autumn period. We noted that whatever method we used for spreading project information, the results in terms of new registrations were substantially lower than in previous clinical trials within the project. For example, influencers with many followers sharing information about the study resulted in fewer new registrations this time compared to our previous studies. A probably hindering factor was that the access to internet-delivered treatment for adolescents in Sweden had generally increased compared to our previous studies 2018–2020. During 2024, several Swedish universities conducted research studies involving free internet-delivered treatment for adolescents with depression. Furthermore, in 2024 a number of healthcare companies had started to offer free psychological treatment online for young people under 18 years with mental health problems. Consequently, the competitive landscape among online treatment providers became markedly more challenging in 2024 compared to earlier periods.

During the second period of recruitment and enrollment August–October, we received 33 registrations from adolescents, which ended in 13 included participants and 22 exclusions. Thus, during the entire recruitment period we received 138 registrations, which resulted in 35 inclusions and 103 exclusions. As enrollment rates gradually declined during the study, despite continuous efforts, we decided to terminate the study recruitment according to time plan in autumn 2024, although we had not reached the desired sample size.

### 3.2 Performance of the algorithm

The present study included a limited sample. In total, 31 patients had both pre- and post-treatment QIDS-A17-SR ratings, allowing classification as either responders or non-responders. The overall non-response rate in this sample was 35% (11/31). By treatment week 3, the model could correctly classify 24 out of 31 patients (77%) as responders or non-responders. However, the model demonstrated higher accuracy in identifying patients with *favorable* treatment outcomes compared to those with *poor* outcomes. Specifically, it correctly identified 95% of responders (specificity) but only 45% of non-responders (sensitivity), yielding an F1 score of 0.59 and a balanced accuracy of 70%.

Of the six patients identified by the model as non-responders by treatment week 3, two were randomized to the adapted treatment arm and three to treatment as usual arm. Both patients in the adapted treatment arm ultimately remained non-responders, as did two of the three patients in the treatment-as-usual group, yielding a positive predictive value of 83% for non-response signals in the sample.

### 3.3 Treatment outcome

For the total sample (*n* = 35), there were significant improvements from pre-treatment to post-treatment regarding depressive symptoms measured using QIDS-A17-SR (*t* (24) = 5.98, *p* < .001), anxiety symptoms measured using GAD-7 (*t* (23) = 4.08, *p* < .001), and emotion regulation measured using DERS-16 (*t* (21) = 4.20, *p* < .001). Within-group effect sizes were large or approaching large on all these measures: QIDS-A17-SR (*d* = 1.08), GAD-7 (*d* = 0.74), and DERS-16 (*d* = 0.79). As sample sizes were extremely small in two of the treatment arms (IPDT as usual at-risk, *n* = 4; IPDT adapted at-risk, *n* = 2), it was not meaningful to use inference statistics to test differences in outcome between treatment arms. However, descriptive data on treatment outcomes for the three treatment groups are presented in Table 2.

**Table 2:** Outcome results.

Measure	Pre, M (SD)	Week 3, M (SD)	Post, M (SD)
QIDS-A17-SR^1^			
IPDT as usual “on track” (*n* = 29)	14.82 (3.34)	9.12 (4.00)	7.60 (5.63)
IPDT as usual “at-risk” (n = 4)	14.25 (4.27)	13.00 (4.00)	10.25 (2.75)
IPDT adapted “at risk” (n = 2)	19.50 (4.95)	15.00 (0)	18.50 (0.71)

GAD-7^2^			
IPDT as usual “on track” (n = 29)	10.66 (4.17)		6.83 (5.23)
IPDT as usual “at-risk” (n = 4)	10.25 (3.77)		5.00 (1.41)
IPDT adapted “at risk” (n = 2)	12.50 (6.36)		14.00 (4.24)

DERS-16^3^			
IPDT as usual “on track” (n = 29)	3.30 (0.71)		2.47 (0.96)
IPDT as usual “at-risk” (n = 4)	3.00 (0.59)		2.86 (0.65)
IPDT adapted “at risk” (n = 2)	3.41 (0.49)		3.96 (0.40)

Notes: 1) QIDS-A17-SR: Quick Inventory of Depressive Symptomatology—Adolescent Self-Report, 2) GAD-7: Generalized Anxiety Disorder 7-item scale, 3) DERS-16: Difficulties in Emotion Regulation Scale 16-item scale.

## 4 Discussion

The present randomized controlled trial aimed to evaluate an adaptive, feedback-informed version of internet-delivered psychodynamic therapy (IPDT) for adolescents with depression. Despite careful planning and extensive recruitment efforts, the study had to be discontinued due to insufficient enrollment. Only 35 participants were included out of the planned 240. Although the limited sample size precludes any firm conclusions regarding efficacy or mechanisms of change, the study provides valuable insights into current challenges of conducting digital psychotherapy research with minors.

The rate of exclusions from screening to randomization in this study (75%) is comparable to the exclusion rates in the previous non-inferiority RCT (73%) (Mechler et al., 2022). The most common reasons for exclusions were also the same: adolescents who could not be reached, who declined participation, who were not depressed, or who already were in treatment. Thus, many redundant screenings might be inevitable to reach a certain sample size in research with depressed adolescents.

The primary reason for termination of the study was the inability to reach adolescents through social media, previously the most effective recruitment channel in earlier ERiCA trials (Lindqvist et al., 2020; Mechler et al., 2022). Recent European Union legislation, including the General Data Protection Regulation (GDPR), the Audiovisual Media Services Directive (AVMSD), and the Digital Services Act (DSA), has introduced restrictions that prevent social media platforms from showing profiling-based advertisements to minors. Consequently, platforms such as Instagram and Facebook no longer allow sponsored posts or targeted content aimed at users under 18 years in the EU.

While these regulations serve an important protective function, they had an unintended but severe impact on recruitment feasibility. Despite extensive dialogue with Meta representatives and attempts to classify study information as non-commercial research communication, posting to minors remained impossible. Alternative methods such as influencer collaborations, outreach to schools, healthcare centers, and youth organizations proved far less effective than social media advertising. These findings illustrate how legislative changes can inadvertently hinder the dissemination of research and the recruitment of underage participants in non-commercial mental health studies.

The recruitment difficulties were likely also compounded by broader changes in Sweden's digital mental health landscape. In 2024, several universities and private healthcare providers offered free internet-based treatments for adolescents, increasing competition for potential participants. While this development is beneficial from a public health perspective, it complicates participant recruitment for randomized trials. Coordinated national strategies may therefore be needed to ensure that innovative studies remain feasible while maintaining access to care for young people.

### 4.1 Clinical and methodological implications

Despite the small sample, participants showed significant pre- to post-treatment improvements in depression, anxiety, and emotion regulation, consistent with previous findings supporting the efficacy of IPDT for adolescents. This suggests that the treatment format remains feasible and potentially effective even under challenging conditions, at least for those depressed adolescents with sufficient motivation to complete diagnostic interviews and engage in treatment.

Another key contribution of the present study was the validation of a predictive algorithm for early identification of participants at risk of non-response. Overall, the algorithm outperformed therapists' predictions in classifying responders and non-responders, demonstrating higher balanced accuracy. However, its ability to detect non-responders was reduced in cases with missing symptom ratings between pre-treatment and treatment week 3. In fact, all but one of the non-responders (*n* = 5) had missing QIDS-A17-SR ratings between these sessions. Specifically, one patient had only a pre-treatment rating, three had ratings at pre-treatment and week 1, and one had ratings at pre-treatment and week 2. In the training dataset, 46 out of 828 observations (5.9%) were missing between these sessions, whereas in the current study 23 out of 140 observations (19.7%) were missing. This difference was statistically significant (Fisher's Exact Test, OR = 3.34, 95% CI [1.86, 5.86], *p* < .001). When symptom ratings are missing, the model defaults more toward the average improvement trajectory observed in the training data. Because most patients improve over the course of treatment, this increases the likelihood of classifying patients as responders when early-session data is incomplete.

In the non-inferiority RCT, adolescents had weekly text chat sessions with their therapist. These chats typically included discussion of recent symptom ratings and likely functioned as both a reminder and a motivational support for completing assessments. In contrast, no such structured weekly chat component was included in the present study. The absence of these regular chats may have reduced adherence to repeated symptom monitoring, thereby increasing the proportion of missing data. These challenges highlight the need for prediction models in general to be robust to realistic levels of missing data in routine clinical settings. Despite this, the mixed-effects modeling approach implemented within a supervised learning framework showed several advantages. First, it allowed direct comparison with conventional machine learning classifiers such as Random Forest and Naïve Bayes, demonstrating comparable or superior performance. Second, it provided interpretable outputs, such as plotted individual symptom trajectories, making the model's decision process more transparent compared to “black box” models. This interpretability is particularly valuable in clinical settings, where understanding model reasoning can facilitate trust and practical application.

Moreover, the model demonstrated a high positive predictive value for non-response signals, suggesting that when the algorithm flagged a case as at risk, this signal was often correct. This indicates potential for future calibration of the model's sensitivity to improve early detection of non-responders.

Moreover, this modeling approach may be suitable for training predictive models on relatively small, clinically representative datasets compared to the sample sizes usually needed for more complex machine learning models. Using mixed models like this could serve as a foundation for future adaptive treatment designs, where early signals of non-response guide personalized intervention adjustments. Feedback-informed and data-driven personalization of digital psychotherapy represents a promising direction for improving treatment outcomes and reducing dropout. However, robust evaluation of such approaches requires sufficiently large samples, which, in turn, depends on feasible recruitment strategies under current regulatory constraints.

### 4.2 Limitations

The most obvious limitation is the small sample size, which precluded the planned between-group comparisons and statistical modeling of change trajectories. The sample may also have been biased toward older adolescents (aged 18–19) who were reachable through social media, potentially limiting generalizability. Moreover, the findings are specific to the Swedish context and to a period of rapid regulatory and infrastructural change, which may limit their applicability to other countries or time frames.

### 4.3 Future directions

To advance digital psychotherapy research with minors, new recruitment infrastructures are urgently needed. Researchers may need to collaborate more closely with schools, youth clinics, municipalities, and national health services to identify and engage eligible participants. Policymakers and research ethics boards should consider mechanisms that allow non-commercial, publicly funded research projects to communicate with minors in compliance with data protection laws.

From a clinical perspective, the encouraging within-group effects and promising predictive accuracy of the feedback algorithm justify continued development of adaptive, feedback-informed IPDT. Future research should focus on integrating predictive algorithms into routine digital care to provide real-time feedback and guide individualized treatment adjustments. Importantly, such integration would allow iterative model refinement based on incoming data, improving both prediction accuracy and clinical utility over time. Future trials should evaluate the impact of these adaptive systems on engagement, treatment response, and dropout in larger, more heterogeneous samples. Future research using data from the present study could include qualitative analyses of potential reasons for adolescents not to complete recruitment process.

## 5 Conclusions

Although the present trial was terminated with insufficient sample size, it offers important lessons for the field of digital mental health. The findings highlight how recent digital advertising restrictions have fundamentally changed the feasibility of recruiting minors for online psychotherapy research. Addressing this structural challenge will be essential for ensuring continued progress in developing accessible, personalized, and evidence-based treatments for adolescent depression. Despite its limitations, the study offers preliminary evidence for a novel predictive approach to treatment response. The validation results suggest that a supervised learning model integrating mixed-effects estimation may help identify patients at risk of non-response and warrants further evaluation against standard predictive modeling techniques including examination of its validity under conditions of missing data.

## Declaration of competing interest

The authors declare that they have no known competing financial interests or personal relationships that could have appeared to influence the work reported in this paper.

## References

[bb0005] Abbass A., Rabung S., Leichsenring F., Refseth J.S., Midgley N. (2013). Psychodynamic psychotherapy for children and adolescents: a meta-analysis of short-term psychodynamic models. J. Am. Acad. Child Adolesc. Psychiatry.

[bb0010] Alaie I., Philipson A., Ssegonja R., Hagberg L., Feldman I., Sampaio F., Möller M., Arinell H., Ramklint M., Päären A., Von Knorring L., Olsson G., Von Knorring A.L., Bohman H., Jonsson U. (2019). Uppsala longitudinal adolescent depression study (ULADS). BMJ Open.

[bb0015] Andersson G., Titov N., Dear B.F., Rozental A., Carlbring P. (2019). Internet-delivered psychological treatments: from innovation to implementation. World Psychiatry.

[bb0020] Bernstein I.H., Rush A.J., Trivedi M.H., Hughes C.W., Macleod L., Witte B.P., Jain S., Mayes T.L., Emslie G.J. (2010). Psychometric properties of the Quick Inventory of Depressive Symptomatology in adolescents: QIDS and adolescents. Int. J. Methods Psychiatr. Res..

[bb0025] Bishop C.M. (2006).

[bb0030] Bjureberg J., Ljótsson B., Tull M.T., Hedman E., Sahlin H., Lundh L.-G., Bjärehed J., DiLillo D., Messman-Moore T., Gumpert C.H., Gratz K.L. (2016). Development and validation of a brief version of the Difficulties in Emotion Regulation Scale: the DERS-16. J. Psychopathol. Behav. Assess..

[bb0035] Cuijpers P., Karyotaki E., Ciharova M., Miguel C., Noma H., Stikkelbroek Y., Weisz J.R., Furukawa T.A. (2021). The effects of psychological treatments of depression in children and adolescents on response, reliable change, and deterioration: a systematic review and meta-analysis. Eur. Child Adolesc. Psychiatry.

[bb0040] Foroushani P.S., Schneider J., Assareh N. (2011).

[bb0045] Hedman-Lagerlöf E., Carlbring P., Svärdman F., Riper H., Cuijpers P., Andersson G. (2023). Therapist-supported Internet-based cognitive behaviour therapy yields similar effects as face-to-face therapy for psychiatric and somatic disorders: an updated systematic review and meta-analysis. World Psychiatry.

[b5000] Hesser H. (2015). Modeling individual differences in randomized experiments using growth models: Recommendations for design, statistical analysis and reporting of results of internet interventions. Internet Interv..

[bb0055] Hiller W., Schindler A.C., Lambert M.J. (2012). Defining response and remission in psychotherapy research: a comparison of the RCI and the method of percent improvement. Psychother. Res..

[bb0060] Lindegaard T., Berg M., Andersson G. (2020). Efficacy of internet-delivered psychodynamic therapy: systematic review and meta-analysis. Psychodyn. Psychiatry.

[bb0065] Lindqvist K., Mechler J., Carlbring P., Lilliengren P., Falkenström F., Andersson G., Johansson R., Edbrooke-Childs J., Dahl H.-S.J., Lindert Bergsten K., Midgley N., Sandell R., Thorén A., Topooco N., Ulberg R., Philips B. (2020). Affect-focused psychodynamic Internet-based therapy for adolescent depression: randomized controlled trial. J. Med. Internet Res..

[bb0070] López-Soler C., Vicente-Escudero J.L., López-López J.A., Alcántara M., Martínez A., Castro M., Fernández V., Sánchez-Meca J. (2024 Jul–Sep). Effectiveness of Internet-delivered psychological treatments for children and adolescents with anxiety and/or depressive disorders: systematic review and network meta-analysis. Int. J. Clin. Health Psychol..

[bb0075] Mechler J., Lindqvist K., Carlbring P., Lilliengren P., Falkenström F., Andersson G., Topooco N., Johansson R., Midgley N., Edbrooke-Childs J., Dahl H.-S.J., Sandell R., Thorén A., Ulberg R., Bergsten K.L., Philips B. (2020). Internet-based psychodynamic versus cognitive behaviour therapy for adolescents with depression: study protocol for a non-inferiority randomized controlled trial (the ERiCA study). Trials.

[bb0080] Mechler J., Lindqvist K., Carlbring P., Topooco N., Falkenström F., Lilliengren P., Andersson G., Johansson R., Midgley N., Edbrooke-Childs J., Dahl H.-S.J., Sandell R., Thorén A., Ulberg R., Bergsten K.L., Philips B. (2022). Therapist-guided internet-based psychodynamic therapy versus cognitive behavioural therapy for adolescent depression in Sweden: a randomised, clinical, non-inferiority trial. Lancet Digital Health.

[bb0085] Mechler J., Lindqvist K., Philips B., Midgley N., Lilliengren P. (2024). Internet-delivered affect-focused psychodynamic therapy for adolescent depression: treatment principles and clinical application in the ERiCA project. J. Infant Child Adolesc. Psychother..

[bb0090] Mörk E., Sjögren A., Svaleryd H. (2014). https://EconPapers.repec.org/RePEc:hhs:ifauwp:2014_008.

[bb0095] Oud M., de Winter L., Vermeulen-Smit E., Bodden D., Nauta M., Stone L., van den Heuvel M., Taher R.A., de Graaf I., Kendall T., Engels R., Stikkelbroek Y. (2019). Effectiveness of CBT for children and adolescents with depression: a systematic review and meta-regression analysis. Eur. Psychiatry.

[bb0100] Rizopoulos D. (2016). The R package JMbayes for fitting joint models for longitudinal and time-to-event data using MCMC. J. Stat. Softw..

[bb0105] Rocha T.B.-M., Graeff-Martins A.S., Kieling C., Rohde L.A. (2015). Provision of mental healthcare for children and adolescents: a worldwide view. Curr. Opin. Psychiatry.

[bb0110] Rozental A., Magnusson K., Boettcher J., Andersson G., Carlbring P. (2017). For better or worse: an individual patient data meta-analysis of deterioration among participants receiving Internet-based cognitive behavior therapy. J. Consult. Clin. Psychol..

[bb0115] Salzer S., Stefini A., Kronmüller K.-T., Leibing E., Leichsenring F., Henningsen P., Peseschkian H., Reich G., Rosner R., Ruhl U., Schopf Y., Steinert C., Vonderlin E., Steil R. (2018). Cognitive-behavioral and psychodynamic therapy in adolescents with social anxiety disorder: a multicenter randomized controlled trial. Psychother. Psychosom..

[bb0120] Sealed Envelope Ltd (2012).

[bb0125] Sheehan D.V., Lecrubier Y., Sheehan K.H., Amorim P., Janvas J., Weiller E., Hergueta T., Baker R., Dunbar G.C. (1998). The Mini-International Neuropsychiatric Interview (M.I.N.I.): the development and validation of a structured diagnostic psychiatric interview for DSM-IV and ICD-10. J. Clin. Psychiatry.

[bb0130] Spitzer R.L., Kroenke K., Williams J.B.W., Löwe B. (2006). A brief measure for assessing generalized anxiety disorder: the GAD-7. Arch. Intern. Med..

[bb0135] Vlaescu G., Alasjö A., Miloff A., Carlbring P., Andersson G. (2016). Features and functionality of the Iterapi platform for internet-based psychological treatment. Internet Interv..

[bb0140] Weersing V.R., Goger P., Schwartz K.T., Baca S.A., Angulo F., Kado-Walton M. (2025). Evidence-base update of psychosocial and combination treatments for child and adolescent depression. J. Clin. Child Adolesc. Psychol..

